# In Type 1 Diabetes a Subset of Anti-Coxsackievirus B4 Antibodies Recognize Autoantigens and Induce Apoptosis of Pancreatic Beta Cells

**DOI:** 10.1371/journal.pone.0057729

**Published:** 2013-02-28

**Authors:** Caterina Bason, Renata Lorini, Claudio Lunardi, Marzia Dolcino, Alessandro Giannattasio, Giuseppe d’Annunzio, Antonella Rigo, Nicoletta Pedemonte, Roberto Corrocher, Antonio Puccetti

**Affiliations:** 1 Department of Medicine, Section of Internal Medicine, University of Verona, Verona, Italy; 2 Department of Pediatrics, University of Genova and Giannina Gaslini Institute, Genova, Italy; 3 Clinical and Experimental Immunology Unit, Giannina Gaslini Institute, Genova, Italy; 4 Department of Medicine, Section of Hematology, University of Verona, Verona, Italy; 5 Laboratory of Molecular Genetics, Giannina Gaslini Institute, Genova, Italy; 6 Department of Experimental Medicine, Section of Hystology, University of Genova, Genova, Italy; La Jolla Institute for Allergy and Immunology, United States of America

## Abstract

Type 1 diabetes is characterized by autoimmune destruction of pancreatic beta cells. The role played by autoantibodies directed against beta cells antigens in the pathogenesis of the disease is still unclear. Coxsackievirus B infection has been linked to the onset of type 1 diabetes; however its precise role has not been elucidated yet. To clarify these issues, we screened a random peptide library with sera obtained from 58 patients with recent onset type 1 diabetes, before insulin therapy. We identified an immunodominant peptide recognized by the majority of individual patients’sera, that shares homology with Coxsackievirus B4 VP1 protein and with beta-cell specific autoantigens such as phogrin, phosphofructokinase and voltage-gated L-type calcium channels known to regulate beta cell apoptosis. Antibodies against the peptide affinity-purified from patients’ sera, recognized the viral protein and autoantigens; moreover, such antibodies induced apoptosis of the beta cells upon binding the L-type calcium channels expressed on the beta cell surface, suggesting a calcium dependent mechanism. Our results provide evidence that in autoimmune diabetes a subset of anti-Coxsackievirus antibodies are able to induce apoptosis of pancreatic beta cells which is considered the most critical and final step in the development of autoimmune diabetes without which clinical manifestations do not occur.

## Introduction

Type 1 diabetes is a chronic autoimmune disease characterized by progressive and selective destruction of pancreatic beta cells in genetically predisposed individuals during childhood or adolescence [1], [2].

As for other autoimmune diseases, it is triggered by the interaction between genetic and environmental factors. Among diabetogenic environmental factors, viruses seem to play a pivotal role as suggested by epidemiological, experimental and clinical data. Enteroviruses, particularly coxsackie B virus infection, have been associated with autoimmunity/type I diabetes [3], [4]. Recent findings have shown that genetic polymorphism of IFIH1 is associated to increased risk to develop type 1 diabetes. This gene encodes for an innate immune system receptor for enteroviruses suggesting one possible mechanism for the diabetogenic effect of enteroviruses. This is further emphasized by the observation that the innate immune system is activated in the pancreatic islets of type 1 diabetic patients [5].

Moreover recent studies show a higher prevalence of enterovirus RNA in serum, plasma or whole blood samples, and in mononuclear cells of newly diagnosed patients with type 1 diabetes than that found among healthy controls [3], [6], [7].

A recent report shows that a large proportion of type 1 diabetic patients have prolonged/persistent enterovirus infection associated with an inflammatory process in the gut mucosa, suggesting that the gut mucosa is a reservoir for enterovirus persistence in patients with type 1 diabetes [8], [9]; however these findings have not been confirmed in another study [10].

Furthermore, coxsackie B viruses have been detected in the pancreatic islets of type I diabetic patients [2], [11], [12]. In some cases infection of pancreatic cells by coxsackievirus B4 has been shown to upregulate the cell surface expression of beta cell proteins, including the autoantigen glutamic acid decarboxylase (GAD), suggesting a role for coxsackievirus B4 in the induction and/or potentiation of autoimmune responses against candidate islets autoantigens [13].

Studies performed in animal models have increased our knowledge on the role of coxsackievirus B4 in type 1 diabetes by helping to clarify the pathogenic mechanisms of the infection that can lead to beta cell destruction, including direct virus-induced beta cell lysis, molecular mimicry, ‘bystander activation’ and viral persistence [14].

Indeed the pathogenic role of viruses in the destruction of beta cells involves direct cell damage, local activation of the immune system and production of proinflammatory mediators, such as cytokines and chemokines that may be detrimental for immune homeostasis in the islets of Langherans and be critical in the pathogenesis of the disease [15]. Other potential pathogenic mechanisms are the impairment of central self-tolerance due to viral infections [16] and the induction of a subset of antibodies able to favour a viral escape from the immune response, thus participating to the spreading of viruses to beta cells [17].

The intervention of cells of the immune system on the pathogenesis of pancreatic damage has been deeply investigated, and the histopathology of type 1 diabetes has been clearly defined by a decreased beta cell mass in association with insulitis, a characteristic lymphocytic infiltration limited to the islets and prominent in the early stage of the disease. A cytotoxic T cell mediated destruction of insulin producing cells is thought to be initiated by an unknown (auto)antigen leading to destruction of beta mass at clinical diagnosis. The infiltrate consists predominantly of T cells, in which CD8+ lymphocytes dominate, but also contains CD4+ lymphocytes, B lymphocytes and a large number of macrophages. On the contrary NK cells are detected rarely even in heavily inflamed islets [18]. The cellular response is accompanied by a humoral response that includes autoantibodies directed against a large array of beta cell antigens. Autoantibodies may precede the onset of type 1 diabetes by months or years and they are mainly considered an epiphenomenon of the tissue damage. In addition little is known on the functional effect of autoantibodies directed against islet cell autoantigens. The aim of this work was to try to clarify some of these issues by using a peptide library approach which we have already successfully applied to the identification of novel disease-specific autoantigen targets in different autoimmune diseases [19]–[23].

The results obtained suggest that coxsackie B4 antibody response may be linked to the pathogenesis of the disease by cross-reacting with autoantigens expressed by pancreatic beta cells, thus identifying a novel property of anti-virus antibodies that may represent an additional mechanism of pancreatic beta cell damage during the pathogenesis of type I diabetes.

## Patients and Methods

### Patients

**Figure 1:**
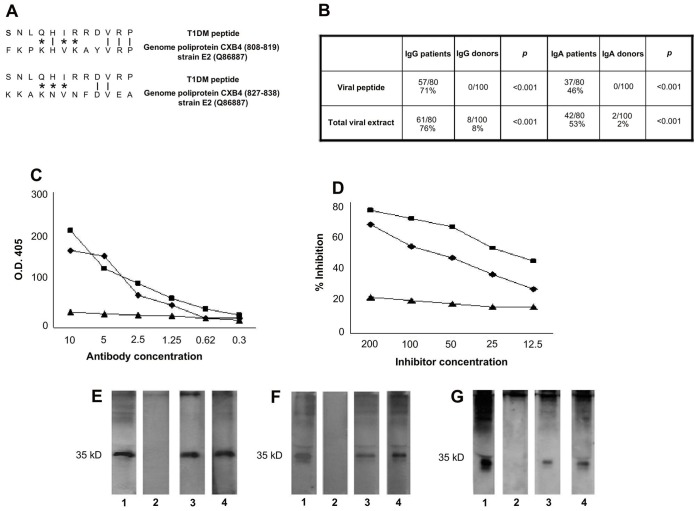
T1DM peptide shares sequence homology with genome poliprotein CXB4 and induces crossreactive antibodies. **A**, Sequence homology between T1DM peptide and the genome poliprotein CXB4, analyzed by the basic local alignment search tool using the National Center for Biotechnology Information (NCBI) network service. Vertical line = identical amino acids; asterisk = conservative substitutions **B**, Frequency of the binding of patients’ sera to COXSA peptide and to total viral extract evaluated by ELISA test. **C**, Direct binding of affinity-purified antibodies against T1DM (black rhombus), COXSA (black square) and irrelevant (black triangle) peptides, to COXSA synthetic peptide. Data represent absorbance at 405 nm (vertical axis) and antibody concentration µg/ml (horizontal axis). **D**, Binding of affinity-purified antibodies against COXSA peptide is inhibited by T1DM (black rhombus), COXSA (black square) peptides but not by an irrelevant (black triangle) peptide. Data represent inhibition percentage (vertical axis) and the inhibitor concentration µg/ml (horizontal axis). **E**, Immunoblot analysis of total viral extract revealed with a commercial positive control IgG (Lane 1) or with antibodies affinity purified against the T1DM peptide (Lane 3) or against the COXSA peptide (Lane 4) or against the irrelevant peptide (Lane 2). **F**, Immunoblot analysis of total viral extract revealed with a commercial positive control IgG (Line 1) or with IgG from a healthy donor’s serum (Lane 2), or with IgG from two different type I diabetes patients’ sera (Lanes 3 and 4). **G**, Immunoblot analysis of total viral extract revealed with a commercial positive control IgA (Lane 1) or with IgA from a healthy donor’s serum (Lane 2), or with IgA from two different type I diabetes patients’ sera (Lanes 3 and 4).

We enrolled fifty-eight patients (35 males, 23 females) aged 0.8–18.7 years (mean age 8.3 years) with recently diagnosed (1–29 days) type 1 diabetes attending the Department of Pediatrics, Giannina Gaslini Institute, Genova. Diagnosis was based on the American Diabetes Association criteria [24]. Patients were tested for anti-glutamic acid decarboxylase (GADA), anti-insulin (IAA) and anti-tyrosine phosphatase-like protein (IA2A) antibodies (Table S1) within 30 days from the diagnosis and before starting insulin therapy. Commercially available kits were used to detect autoantibodies (Radioimmuno-assay CIS Bio International-Schering SA). One hundred age and sex-matched healthy individuals served as controls. Eight subjects with evidence of Coxsackievirus infection and presence of serum antibodies directed against the Coxsackievirus B4 extract were tested for the presence of antibodies against the T1DM and COXSA peptides (Fig. 1B).

A second group of twenthy-two subjects (13 males, and 9 females) aged 2–15 years (mean age 9.1 years) with recent onset type I diabetes was also tested to validate the results.

Written informed consent was obtained from children’s parents and from patients and controls who were older than 18 years. The institutional review board of Giannina Gaslini Institute approved the research. All clinical investigations have been conducted according to the principles expressed in the Helsinky declaration.

### Cell Lines

Since human and murine autoantigens shares high degree of homology (Table S2), we used the pancreatic beta-cell line B-TC1 (a kind gift of F. Dotta, University of Siena, Italy) between passages 3–9 and the beta-cell line NIT-1 (ATCC n°CRL-2055; Rockville, MD, USA) between passages 3–12. B-TC1 cells were cultured in D-MEM with 15% horse serum and 2,5% heat-inactivated fetal calf serum and 1% penicillin-Streptomycin purchased from GIBCO (Invitrogen, Carlsbad, CA, US). NIT-1 cells were cultured according to the recommendations of ATCC in F-12 Ham’s medium (FK12) containing 10 mmol/L d-glucose supplemented with sodium bicarbonate and 10% head-inactivated fetal calf serum (GIBCO-Invitrogen, Carlsbad, CA, US).

### Library

The FliTrx random dodecamer peptide library was purchased from Invitrogen (Carlsbad, CA, USA) and screened with pooled Igs affinity-purified from the sera of 58 patients with type 1 diabetes as described elsewhere [19]–[22].

Each ‘biopanning’ experiment was preceded by a ‘pre-panning’ step with pooled normal IgG obtained from 50 normal donors to remove bacteria that nonspecifically bind immunoglobulins outside the antigen-binding site. After five sequential rounds of ‘biopanning’ experiments, the enriched library was grown, and single colonies were picked, expanded and induced with tryptophan to express the fusion peptides. Bacteria were then lysed in sample buffer and tested by western blot analysis with the pooled T1DM IgG fraction to find positive clones. DNA was extracted from positive clones and sequenced. A set of 15 out of 31 peptides obtained from the last biopanning experiment, was synthesized and used to test sera from type 1 diabetes patients and controls in ELISA.

### Peptide Synthesis

All the synthetic peptides, including T1DM-peptide (SNLQHIRRDVRP), COXSA-peptide (FVPKHVKAYVRP), CA-peptide (GNLEHVS) and the irrelevant control peptide (VTAPKDSDVELP), were manually synthesized [25].

### ELISA

The direct and competitive ELISA method for antibody binding to the synthetic peptides has been described elsewhere [19]–[22]. The synthetic peptides were used at the concentration of 20 µg/ml in PBS to coat polystyrene plates (Immulon II, Dynax, Ashford, UK).

As secondary antibodies, we used anti-human IgG antiserum (Sigma, Saint Louis, MO, USA) or anti-human IgA antiserum (Sigma, Saint Louis, MO, USA). Absorbance values higher than the mean plus three standard deviation of each serum dilution of the control group were considered positive. Anti-Coxsackievirus antibodies (IgG and IgA) in type I diabetes patients and healthy donors was assessed using a total Coxsackievirus B4 estract (Virion, Ruschlikon, Switzerland) (25 µg/ml in PBS).

### Western Blot

The Coxsackievirus B4 extract was used to detect the VP-1 protein.

The virion proteins were resolved by SDS-PAGE 10% and transferred to nitrocellulose membrane (Amersham Bioscience, Piscataway, New Jersey, USA). Blots were probed with primary antibodies followed by either peroxidase-linked anti-human IgG (Amersham Bioscience, Piscataway, NJ, USA) or anti-human IgA (Sigma, Saint Louis, MO, USA). Positive controls sera (IgG/IgA against Coxsackievirus) were supplied by Virion. The VP1 protein was identified with commercially available antibodies (Acris Antibodies, Inc, San Diego, CA, USA).

### Immunoprecipitation

Beta cells (1×10^7^) were lysed in cold mild detergent conditions (1% Nonidet P- 40, 10 mM Tris, pH 7.4, 0.15 M NaCl and 5 mM MgCl_2_) (Sigma, Saint Louis, MO, USA) and immunoprecipitated with affinity-purified antibodies coupled to sepharose–protein A (Pharmacia Biotech, Piscataway, NJ). Eluted proteins were separated by discontinuous SDS–PAGE (10% for IA-2β, 8% for PFKP, 6% for CACNA1D) and transferred to nitrocellulose membrane. For detection we used the following antibodies: 1) anti-IA-2β chicken antibody (GenWay Biotech, San Diego, Ca, USA), 2) anti-PFKP rabbit antibody (Abgent, San Diego, CA, USA) and 3) anti-CACNA1D rabbit antibody (Sigma, Saint Louis, MO, USA). The secondary antibodies used were goat Anti-IgY Antibody HRP Conjugate (Gen Way-San Diego) and anti-rabbit IgG HRP Conjugate (Amersham Bioscience, Piscataway, New Jersey, USA) respectively. The ECL Plus Western Blot Detection System kit (Amersham, Bioscience, Piscataway, New Jersey, USA) was used for detection.

### FACS Analysis

B-TC1 cells were surface stained with primary anti-IA-2β chicken antibody followed by PE-tagged anti-chicken antibody (Jackson Immunoresearch). For the competitive assay beta cells were first preincubated with anti-T1DM or COXSA antibodies (37°C for 30 min) then washed and incubated with anti-IA-2β chicken antibody (4°C for 30 min) and washed and stained with PE-tagged anti-chicken antibody (Jackson Immunoresearch).

B-TC1 cells, fixed and permeabilized, (Fix &Perm, Caltag, AT), were incubated with primary anti-PFKP rabbit antibody (Abgent, San Diego, CA, USA) followed by PE-labelled anti-rabbit antibody (Jackson Immunoresearch).The competitive assay was carried out as follows: B-TC1 cells were first incubated with primary anti-PFKP rabbit antibody then washed and stained with anti-T1DM or anti-COXSA peptide antibodies with followed by anti-human PE-coniugated antibodies (Jackson Immunosearch). Data were acquired on FACSCalibur and analyzed with Flow Jo 8.8.2 software. Results are expressed as mean fluorescence intensity (MFI).

### Measurement of Apoptosis

Internucleosomal DNA fragmentation was quantified using a commercially available kit (Roche Biochemical, Indianapolis, I, USA). Beta- cells (1×10^5^cells/ml) were cultured for 24 hours in the presence or absence of anti- T1DM, anti-COXSA, anti-CA peptides antibodies affinity-purified from patients’ sera. As positive control cells were cultivated in presence of apoptotic stimuli (50 ng/ml TNF-α or 10 µg/ml cycloheximide).

### Mitochondrial Membrane Potential Evaluation

Beta cells were incubated either with anti-T1DM peptide antibody or with medium alone for 6 hours at 37°C, washed and exposed to potential-sensitive-dye 5,5′,6,6′-Tetrachloro-1,1′,3,3′-tetra-ethylbenzimidazolocarbocyanine iodide (JC-1) (Molecular Probes, Eugene, OR, USA) to detect changes in mithocondrial transmembrane potential (DΨ_m_). Campothecin (CCCP) (Molecular Probes) which causes quick mithocondrial membrane depolaritazion, was used as positive control. Cells were recorded on a FACSCalibur cytometer (Becton Dickinson, San Jose, CA, USA). Emission of JC-1 monomers was detected in Fl-1 using a 530/30 nm bandpass filter, and JC-1 aggregates were detected in Fl-2 using a 585/42 nm bandpass filter. FlowJo 8.8.2 software (Tree Star, Ashland, OR) was used to analyze the data [26], [27]. Beta cells were seeded onto a slide and incubated at 37°C for 6 hours. After staining with JC-1 they were recorded by an Axio Observer inverted microscope (Zeiss, Gottingen, DE). Visualization of JC-1 monomers (green fluorescence) and JC-1 aggregates (red fluorescence) was carried out using filter sets for fluorescein and rhodamine dyes (emission 488 and 550 nm respectively). Image analysis was performed using Axiovision 3 software.

### Intracellular Calcium Measurement

Beta cells (4×10^4^cells/well) were plated on 96-well black wall/clear plate (Costar, Lowell, MA, USA). The following day, cells were washed and incubated with a loading solution containing the fluorescent probe FluoFORTE™ (Enzo Life Sciences, Plymouth Meeting, PA, USA) and sulfinpyrazone (200 µM). After 1 hour, TNFα (50 ng/ml), the anti-T1DM antibody (20 µg/ml) or PBS were added to cells. The plate was then transferred to the microplate reader (excitation: 485 nM; emission: 520 nM). Each assay consisted in a 2-sec fluorescence reading after which a high-potassium solution was injected into the well (final K+ concentration 90 mM). The fluorescence was monitored for additional 48 sec. The increase of intracellular calcium concentration was determined as the increase in FluoFORTE fluorescence [28].

### Statistical Analysis

The frequencies of antibodies directed against the peptide and the viral extract between type 1 diabetes patients and healthy donors were performed using the χ square Pearson’s test. Differences in intracellular calcium concentration were evaluated using the non-parametric Mann-Whitney test.

## Results and Discussion

### Detection of Anti-beta Cell Autoantibodies in the Patients’sera

The sera of the patients used to screen the peptide library, were also tested for the presence of autoantibodies typical of type 1 diabetes. Anti-Glutamic acid decarboxylase (GAD) antibodies were positive in 44/58 of patients (75,9%), anti-transmembrane protein tyrosine phosphatase (IA-2) antibodies were positive in 43/58 (74,1%) and anti-proinsulin/insulin (IAA) antibodies in 12/58 (20,7%) (Table S1).

### Screening of the Random Peptide Library and ELISA for the Identification of Relevant Antigens

To define pathogenetically relevant autoantigens in type 1 diabetes, we screened a dodecamer random peptide library [19]–[23], with pooled immunoglobulins derived from 58 patients with recent onset type I diabetes before insulin therapy. A set of 15 peptides, out of the 31 peptides obtained from the last biopanning round, was synthetized and used to test the individual patients’sera in a direct enzyme-linked immunosorbent assay (ELISA) employing the solid phase peptide. By this approach, we identified a peptide called T1DM peptide (SNLQHIRRDVRP) that was recognized by serum IgG of 76% (44/58) individual patients, by both direct ELISA (absorbance mean plus/minus s.d. : 0.245 plus/minus 0.029 for a serum dilution of 1∶200) and competitive ELISA; such reactivity was not detected in the sera of 50 age- and sex-matched healthy controls (absorbance mean plus/minus s.d.: 0.047 plus/minus 0.022). We also tested the sera for the presence of IgA antibodies against the identified peptide and we observed that these antibodies were detectable in 45% (26 of 58) patients, but not in the control group. To validate these data, another group of 22 patients with early onset type 1 diabetes, whose sera were not used to screen the library, was analysed: 16/22 individuals (73%) had serum IgG against the identified peptide, and 10/22 subjects (45%) had serum IgA against the T1DM peptide.

Altogether IgG antibodies were present in 60/80 (74,5%) patients’sera and IgA antibodies were detectable in 36/80 (45%) sera.

These data indicate that this T1DM peptide sequence contains an epitope recognized by the majority of the sera of patients with type 1 diabetes.

### Anti-T1DM Peptide Antibodies Recognize the Coxsackie Derived VP1 Protein

Since enteroviruses, in particular type B coxsackieviruses, have been associated with type 1 diabetes on the basis of epidemiological, clinical and experimental observations [5], [8], [11], [15], [29]–[33], we compared the amino-acid sequence of T1DM peptide with known viral sequences in a protein data bank (Swiss-Prot database) using the BLASTP via the NCBI BLAST network service. We found that the peptide shared homology with different proteins including coxsackievirus B4 (CVB4) genome poliprotein CXB4 of the diabetogenic strain E2. The homology resides in two different areas of the molecule contained within the VP1 protein (aa 569–849), a protein expressed on virus capside (Fig. 1A) [33]. Based on the extent of homology (number of matched amino acids, both identities and conservative substitutions) we synthetized the viral peptide 808–819 of strain E2 and used it in an ELISA assay to test patients’sera. Such peptide (called COXSA peptide: FKPKHVKAYVRP) was recognized by the majority of individual patients’sera. Indeed 57 out of 80 patients' sera (71%) had IgG antibodies against the peptide, while IgA antibodies were present in 37 out of 80 patients’ sera (46%). None of the controls had IgG or IgA antibodies against the peptide (p<0.001) (Fig. 1B). Moreover 6 of the 80 patients’sera had only IgA antibodies directed against the COXSA peptide, while 17/80 had neither IgG or IgA antibodies against the peptide. Interestingly the same 17 patients did not have either IgG or IgA antibodies against the T1DM peptide.

We next evaluated whether the patients’sera recognized also the whole viral extract and found that the frequency of antibodies directed against the COXSA peptide is similar to the frequency of serum IgG and IgA antibodies directed against a commercially available CVB4 extract. Sixty-one out of 80 patients (76%) had IgG antibodies against CVB4 extract in ELISA, while 42 out 80 (53%) patients had IgA antibodies against the viral extract (Fig. 1B). One patient had only IgA antibodies against the CVB4 extract. The remaining 17 patients, whose serum Igs did not bind the viral extract, are the same who did not recognized the T1DM and COXSA peptides.

We next purified antibodies directed against T1DM and COXSA peptides from individual sera of ten patients by affinity chromatography using peptide-Sepharose columns. These affinity purified antibodies bound the COXSA peptide in ELISA (Fig. 1C). Moreover, the binding of the purified antibodies to the COXSA peptide was competed by both peptides, but not by an irrelevant control peptide (Fig. 1D).

The affinity-purified antibodies revealed a band of about 35 kDa in western blot, using the total CVB4 extract (Fig. 1E). This molecular weight is compatible with the VP1 coxsackievirus capsid protein. IgG and IgA antibodies directed against the identified protein were detected in western blot also in individual patients’sera at a frequency similar to the one observed in ELISA (80% and 55% respectively). Noteworthy patients’sera reacted almost exclusively with the 35 kDa protein (Fig. 1F and G) and this reactivity was never detected in controls’ sera. It is interesting to note that the capsid protein VP1 has been recently shown to be present in beta cells of more than 60% of patients with recent-onset diabetes and in very few age-matched controls. VP1 expression correlates with other markers of viral infection, consistent with the presence of active virus in the islets cells of patients with type 1 diabetes [13].

These data indicate that the presence of anti-Coxsackievirus B4 serum antibodies is a feature of type I diabetes and that in particular the COXSA peptide contains a crucial epitope of the anti-Coxsackievirus antibody response in patients with type 1 diabetes suggesting another possible link between coxsackie B4 virus infection and type 1 diabetes.

### Anti-T1DM and Anti-COXSA Peptides Antibodies Recognize Beta Cell Antigens

**Figure 2:**
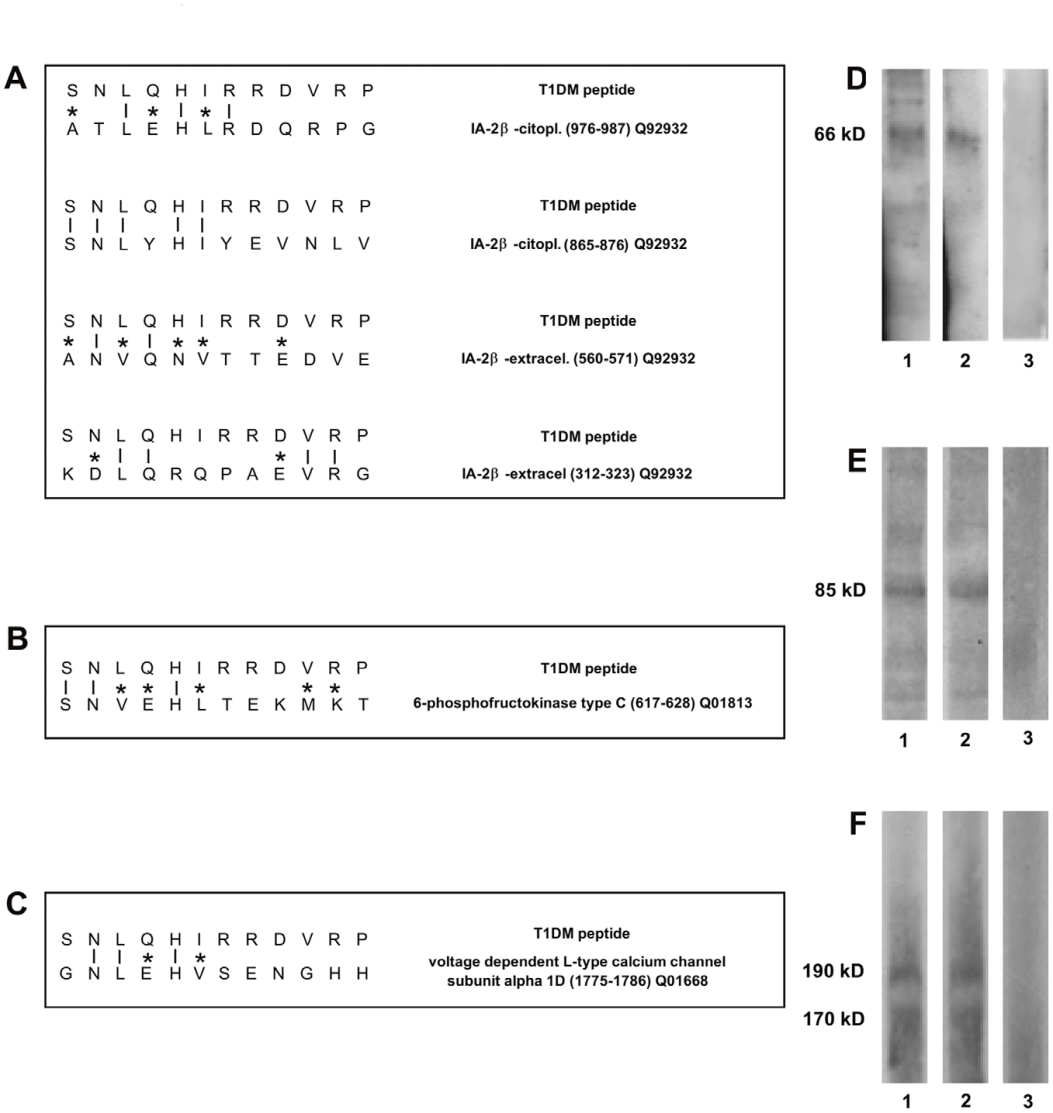
Antibodies against the T1DM and COXSA peptides bind autoantigens. **A**, **B**, **C**, T1DM peptide shares homology with IA-2β, 6-PFKP, CACNA1D autoantigens respectively; vertical line = identical amino acids; asterisk = conservative substitutions. **D**, Immunoblot analysis of beta cells lysates immunoprecipitated with affinity purified antibodies against T1DM peptide (Lane 1) or against COXSA peptide (Lane 2) or against the irrelevant peptide (Lane 3) revealed with specific anti-phogrin antibody, or **E**, with specific anti-PFKP antibody, or **F**, with specific anti-L-type calcium channel antibody: the two bands represent different isoforms of the CACNA1D molecule.

**Figure 3:**
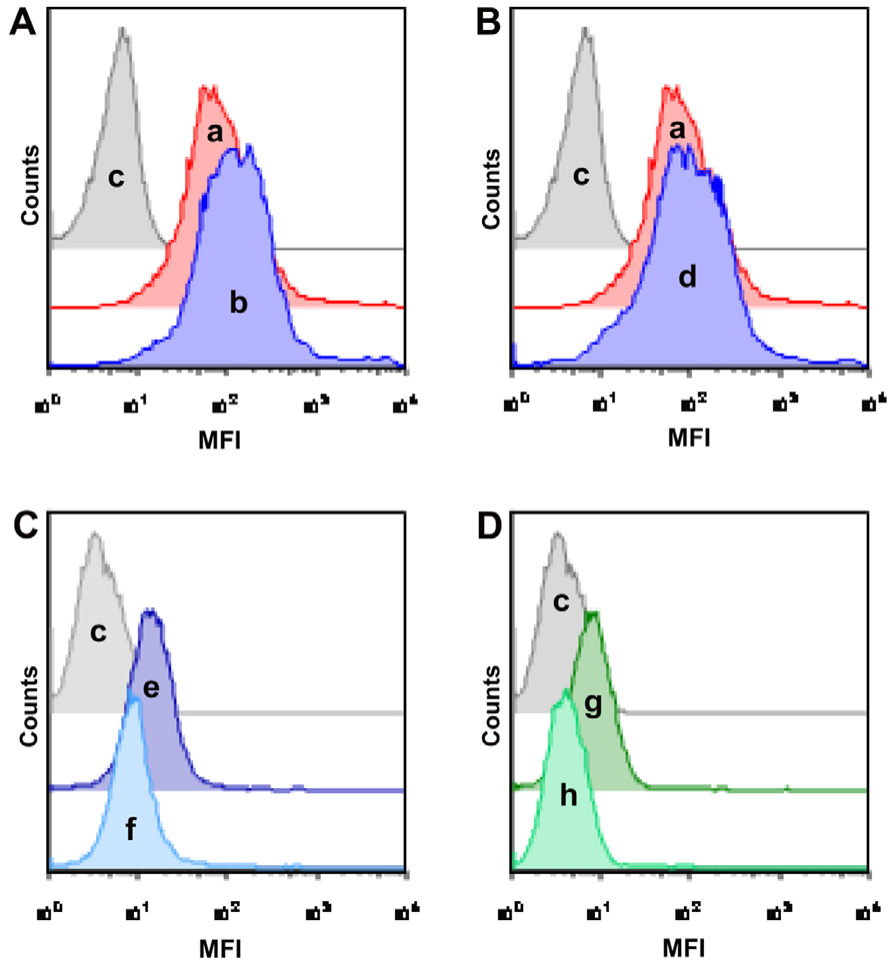
Anti-peptides antibodies bind cell surface phogrin and intracellular PFKP. Preincubation of beta cells with anti T1DM peptide antibodies **(A)**, and with anti COXSA peptide antibodies (**B**) resulted in an increase of the binding of anti-phogrin antibodies a = anti-IA-2β chicken antibody and secondary PE-tagged anti-chicken antibody; MFI = 111 b = anti-T1DM peptide antibodies followed by anti-IA-2β chicken antibody and by secondary PE-tagged anti-chicken antibody; MFI = 162 d = anti-COXSA peptide antibodies followed by anti-IA-2β chicken antibody and by secondary PE-tagged anti chicken antibody; MFI = 130 c = control; MFI = 6. Preincubation with anti-PFKP rabbit antibody reduced the binding of anti-T1DM peptide antibodies (**C**) and anti-COXSA peptide antibodies (**D**) to intracellular PFKP. e = anti-T1DM peptide antibodies and secondary anti-human PE-coniugated antibody, MFI = 16.6 f = anti-PFKP rabbit antibodies followed by anti-T1DM antibodies and secondary anti-human PE-coniugated antibody; MFI = 10.4 g = anti-COXSA peptide antibodies and secondary anti-human PE-coniugated antibody; MFI = 9 h = anti-PFKP rabbit antibodies followed by anti-COXSA peptide antibodies and secondary anti-human PE-coniugated antibody; MFI = 4,6 c = control; MFI = 4.1Representative example of three independently performed experiments that generated similar FACS profiles. x-axis: MFI = Mean Fluorescence Intensity; y-axis: cell counts.

As type 1 diabetes is characterized by autoimmune injury of the pancreatic beta insulae, we next compared the T1DM peptide sequence with human proteins in a protein data bank and found that the peptide shares homology with 3 different beta-cell specific self-antigens: 1) IA-2β, also called phogrin (PTPRN2) (Fig. 2A), 2) the enzyme 6-phosphofructo-1-kinase (PFKP) (Fig. 2B), 3) voltage-dependent L-type calcium channel alpha-1D subunit (CACNA1D) (Fig. 2C).

IA-2β, also called phogrin (PTPRN2) [34]–[38] is an integral membrane glycoprotein localized to dense-core secretory granules in various neuroendocrine cells including pancreatic beta cells. The molecule colocalizes with insulin on secretory granules and translocates to the plasma membrane whenever insulin exocytosis is induced by glucose. Phogrin is an important target of autoantibodies in 50% of newly diagnosed type I diabetes patients and antibodies to such molecule can be present years before clinical onset of diabetes. Phogrin expression in pancreatic beta cells is induced by glucose, insulin and cAMP-generating agents, whereas proinflammatory cytokines such IL1β, TNFα and IFNγ cause a down regulation of phogrin levels. Phogrin expression appears to be concomitant with the development of cellular secretory responses [36].

Recent studies show that phogrin contributes to pancreatic beta cell growth by interacting with insulin receptor and by regulating stability of IRS2 protein (insulin receptor substrate 2). The authors propose that phogrin functions as an essential regulator on insulin action in beta cells [39].

The enzyme 6-phosphofructo-1-kinase (PFKP) is a cytoplasmic enzyme with complicated allosteric kinetics that catalyzes a major rate-limiting step of glycolisis, and allows the conversion of fructose 6-phosphate to fructose 1,6-diphosphate. It is tightly regulated by a large variety of metabolites, drugs and intracellular proteins. Also hormones such as insulin, serotonin and epinephrine control PFKP activity through regulatory phosphorylation at critical amino acid residues [40], [41]. One of the most potent activators of PFKP is fructose 2,6 bisphosphate (F2-6BP) and its cellular levels are correlated with glycolytic flux [42]. Noteworthy the protein-coding sequence of human PFK-c has been cloned from pancreatic islet and its deficiency causes impaired insulin secretion and insulin resistance typical of Type 2 diabetes [43], [44].

Voltage-dependent L-type calcium channel alpha-1D subunit (CACNA1D) is involved in a variety of calcium-dependent processes. In pancreatic beta cells the L-type voltage-gated calcium channels (VGCC) mediate the entry of calcium ions leading to secretion of insulin [45], [46].

Hyperactivated VGCC-mediated Ca^++^ overload can induce beta cell apoptosis through various calcium-sensitive enzymes e.g. calcineurine, calpains, endonucleases etc as reported in different papers [47], [48].

Noteworthy the three identified human autoantigens share an high degree of homology with the murine counterparts (respectively 79% for phogrin, 95% for 6PKFP and 97% for CACNA1D) (Table S2A). In particular the regions of homology identified by the T1DM peptide in the human molecules are nearly identical to the murine ones (Table S2B). For this reason we could use murine pancreatic beta cells in our experiments.

We then investigated whether the purified antibodies against the T1DM and COXSA peptides were able to recognize the IA-2β, 6-PFKP and CACNA1D molecules. We observed that such antibodies, but not the anti-irrelevant peptide antibodies, were able to immunoprecipitate the three molecules in beta cells lysates. Indeed, we identified a band of approximately 66 KDa corresponding to phogrin (Fig. 2D); a band of 85 KDa corresponding to 6-PFKP (Fig. 2E); and two bands of 170 and 190 KDa corresponding to L-type Ca2+ channels alfa-1D subunit (Fig. 2F). These two bands represent two different size isoforms of the CACNA1D molecule as already reported by other investigators [49].

Specific binding of the anti-peptide antibodies to the three identified autoantigens was confirmed by mass spectrometry.

We also found that the antibodies against the peptides were able to bind IA-2β expressed on the surface of beta cells (Fig. 3A and B) and the intracellular PFKP (Fig. 3C and D) by FACS analysis.

Interestingly we observed that exposure of beta cells to anti-peptide antibodies resulted in upregulation of cell surface phogrin. The specific mechanisms which regulate this phenomenon are beyond the scope of this study. Torii S. [39] has recently shown that antibodies directed against phogrin induce translocation of the molecule from the storage granules to the cell membrane therefore inducing an increased expression of phogrin during exocytosis.

Based on this report we can speculate that our antibodies may act similarly.

Taken together, these data show that the anti-T1DM and anti-COXSA peptides antibodies specifically recognize autoantigens expressed by pancreatic beta cells.

### Purified Anti-peptide Antibodies and Patients’ Serum Recognize a Specific Epitope of CACNA1D

L-type voltage-gated calcium channels (VGCC) play a key role in pancreatic beta-cell pathophysiology [45], [46]. In particular the identified beta cell antigen CACNA1D belongs to the calcium channel alpha-1 subunit family. In most cases, activation of this subunit is sufficient to generate voltage-sensitive calcium channel activity.

**Figure 4:**
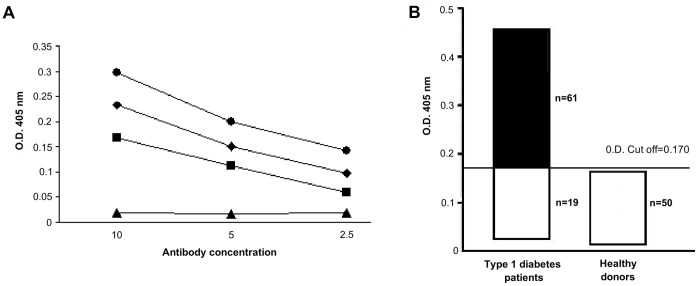
Specific binding to CA synthetic peptide. **A**, direct binding of affinity purified antibodies to T1DM ((black rhombus), COXSA (black square), CA (black circle) or irrelevant (black triangle) peptides to CA synthetic peptide. Data represent absorbance at 405 nm (vertical axis); antibody concentration µg/ml (horizontal axis). **B**, Graphical representation of type I diabetes patients’ or controls’ sera binding to CA synthetic peptide.

To better characterize the interaction between our antibodies and CACNA1D we synthetised a seven aa peptide (GNLEHVS, called CA peptide) derived from CACNA1D and sharing homology with the T1DM peptide. We firstly verified that the peptide was recognized by antibodies purified against the T1DM peptide, the COXSA and the CA peptides in ELISA (Fig. 4A). Secondly we tested whether the peptide was recognized by the sera of patients with type I diabetes. Sixty-one out of 80 (76%) patients’sera had IgG antibodies against the CA peptide in ELISA, whereas none of the 50 controls’sera bound the peptide (Fig. 4B). These results indicate that anti-peptide antibodies react with particular type of VGCC, CACNA1D and suggest that CACNA1D may represent an important and yet unidentified autoantigen target in type I diabetes.

### Anti-peptides Antibodies Induce Beta Cells Apoptosis

Beta-cell apoptosis leading to progressive loss of pancreatic beta cells is a crucial step in the development of type I diabetes [1].

In the early stages of the disease infiltrating macrophages and T cells release proinflammatory cytokines (IL1-β, TNFα, and IFN-γ) which together with cell-to-cell death factors (granzyme B, FasL etc) contribute to the induction of beta-cell apoptosis and the buildup of insulitis [50]–[52].

Some reports suggest a role for Ca2+ in cytokine-mediated pancreatic beta-cell death [53]. Interestingly, sera from patients with type 1 diabetes have been shown to promote calcium-induced apoptosis of beta cells upon activation of VGCC.

Similarly, elevated serum levels of apolipoprotein III are involved in hyperactivated VGCC-mediated beta cell apoptosis [47].

**Figure 5:**
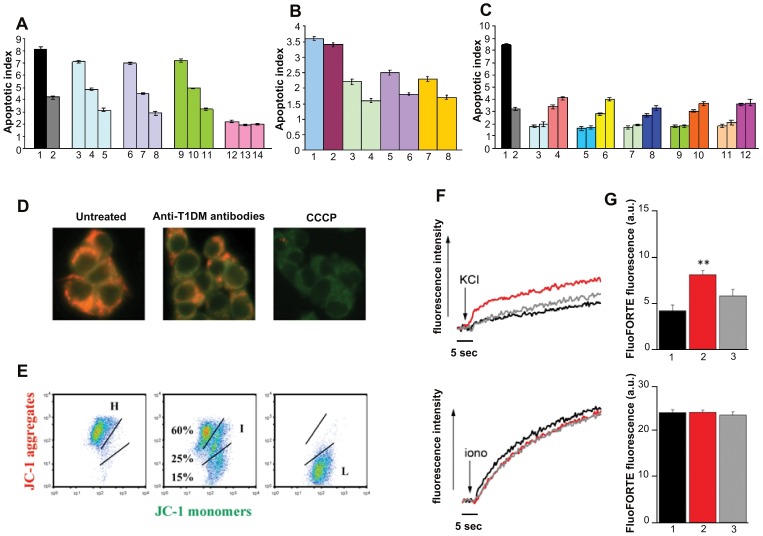
Antibodies against T1DM, COXSA, CA peptides induce apoptosis in beta-cell line through disruption of mitochondrial membrane potential and increase of intracellular calcium. A, NIT cells were incubated for 24 hours with antibodies against: T1DM peptide (bar 3,4,5), COXSA peptide (bar 6,7,8), CA peptide (bar 9,10,11), irrelevant peptide (bar 12,13,14), at three different concentrations (40, 20, 10 µg/ml) or incubated with cycloheximide (bar 1)(10 µg/ml) or TNF-α (bar 2)(50 ng/ml). The apoptotic index, reported in the vertical axis as mean ± s.d. of triplicates, shows the enrichment of nucleosomes released in the cytoplasm (value in control untreated cells = 1). An increase in the enrichment factor of 1.0 corresponds approximately to 10–12% of apoptotis. B, Apoptosis in NIT cells induced by affinity purified antibodies against T1DM peptide 10 µg/ml (bar 1) is inhibited by preincubation of these antibodies with T1DM peptide (bar 3,4), COXSA peptide (bar 5,6) or CA peptide (bar 7,8) at two different concentrations (100, 200 µg/ml) but not by the preincubation with the irrelevant control peptide (bar 2) at the maximum concentration (200 µg/ml). C, Induction of apoptosis of NIT cells by serum of five type I diabetes patients (bar 4,6,8,10,12) or by five controls sera (bar 3,5,7,9,11) at two different dilutions (1∶100, 1∶200) or by cycloheximide (bar 1)(10 µg/ml) or TNFα (bar 2) (50 ng/ml). Sera were heat-inactivated (56°C for 30 min). Data represent the mean ± s.d. of triplicate samples of three independent experiments. D, E, Anti-T1DM antibody alter the mitochondrial membrane potential (DΨ_m_) of beta cells: D, *Microscopy* (magnification x40)*:* Untreated beta cells (negative control) with well-polarized mitochondria are characterized by punctated red fluorescence (left slide); cells treated with anti-T1DM antibody show a population with intermediate mitochondrial transmembrane potential with a partial or complete loss of red fluorescence that is replaced by diffuse green fluorescence (center slide). Cells treated with CCCP (positive control), that causes fast mitochondrial membrane depolarization, show a diffuse green fluorescence (right slide). E, *Flow cytometry.* Untreated cells with well-polarized mithocondria are localized in the upper region of the plot (H = high DΨ_m,_ FL2 ^bright^). Cells exposed to anti-T1DM antibody for 6 hours shift downward (I = intermediate DΨ_m_ and L = low DΨ_m,_ FL2 ^dim^). This shift represents the progressive loss of mitochondrial JC-1 aggregates and an increase in the formation of green fluorescent cytoplasmic monomers, which indicates the disruption of the mitochondrial DΨ_m_. Cells treated with CCCP completely shift downward (L = low DΨ_m,_ FL2 ^dim^). F, G, Anti-T1DM antibody induce intracellular calcium mobilization: F, representative fluorescent traces showing the response to K^+^ addition in beta cells. Prior to assay, beta cells were stimulated with PBS alone (control; black), TNFα (50 ng/ml; gray), or the anti-T1DM antibody (20 µg/ml; red). The curves show the response of cells acutely treated with KCl alone (top panel) or in combination with ionomycin (1 µM; bottom panel). G, Bar graphs summarizing the results of experiments shown in panel F. The intracellular calcium increase was determined as the fluorescence mean triggered by KCl alone (top panel) or in combination with ionomycin (1 µM; bottom panel) in beta cells treated with PBS alone (control; black), TNFα (gray), or the anti-T1DM antibody (red); (mean ± S.E.M., n = 6). **, P<0.01 versus control.

Based on these observations we decided to verify whether affinity-purified antibodies against the peptides had any functional effect on beta cells. To this aim we incubated beta cells for 24 hours with antibodies against the T1DM, COXSA, CA peptides and evaluated the ability of these antibodies to induce apoptosis. Indeed antibodies directed against the three peptides were able to induce internucleosomal DNA fragmentation in beta cells in a dose-dependent fashion reaching the maximum at an antibody concentration of 40 µg/ml (Fig. 5A and Fig. S1). Apoptosis was inhibited by a 1 hour preincubation of peptide-specific antibodies with each of the three peptides (Fig. 5B), further confirming the specificity of antibodies-beta-cell interaction. Moreover, patients’sera, incubated with beta cells for 24 hours, were able to induce beta-cell apoptosis (Fig. 5C).

We next investigated the possible mechanism of beta-cell apoptosis induced by purified anti-peptide antibodies, by measuring both mitochondrial membrane permeabilization and intracellular calcium concentration.

The participation of Ca2+ in beta-cell death appears to have a close relevance to the mitochondrial events or endoplasmic reticulum (ER) stress that represent an important step of the cell death machinery recently identified [54].

Ca2+ is also a prototypic agent inducing mitochondrial permeability transition [55], which has been considered as the ‘point of no return’ in several cell death models.

To detect changes in mitochondrial transmembrane potential (DΨ_m_), we analysed the red/green (aggregates/monomers) ratio of fluorochrome JC-1 on beta-cell by microscopy and flow cytometry [26], [56]. We observed that anti-T1DM antibodies cause mithocondrial membrane depolarization, characterized by loss of JC-1 aggregates and increased formation of monomers with replacement of punctate red by diffuse green fluorescence, already after 6 hours of incubation (Fig. 5D and E), showing that such antibodies are able to induce a mithocondrial membrane permeabilization, hallmark of apoptosis [27]. Interestingly, exposure of beta cells to ds-RNA derived from Coxsackieviruses has been shown to promote beta cells apoptosis by activating the mithocondrial pathway [57].

To assess the effect of the same antibodies on Ca^2+^ channels, we measured intracellular calcium in beta cells using the Ca^2+^-sensitive probe FluoFORTE. A rapid addition of a high-K^+^ solution triggers membrane potential depolarization and Ca^2+^ channel activation. The results obtained show that in the presence of the anti-T1DM antibody, the Ca^2+^ increase triggered by K^+^ addition, was significantly higher (p<0.01) when compared to untreated cells (Fig. 5F). On the contrary, the response triggered by ionomycin, a Ca^2+^ ionophore, was unaltered in treated and untreated cells (Fig. 5G), indicating that the antibody does not modify the cell calcium stores. These data show that anti-T1DM peptide antibodies activate VGCC, resulting in increased intracellular calcium, suggesting that the mechanism at the basis of antibody-induced beta cells apoptosis may be Ca++ dependent.

Autoantibodies directed against VGCC have been already described in type 1 diabetes and have been shown to mediate autonomic dysfunction of the gastrointestinal tract and bladder. Passive transfer experiments have shown that anti-VGCC antibodies from patients with diabetes exert an agonistic effect on L-type VGCC of smooth muscle, resulting in altered contraction during parasympathetic activity. In another study Jackson M.J. et al. [58] showed that such antibodies were able to modify cell functions of rat insulinoma beta cells Rin A12 upon engagement of VGCC. Indeed they showed that these antibodies reversibly decrease impedance of Rin A12 cells upon binding VGCC and that this effect is likely to represent a lack of cell adhesion, derived from changes in cell attachment or spread. The authors conclude that in the absence of additional signals the aberrant calcium channel activity induced by their functional anti-VGCC antibodies may be insufficient for the induction of the apoptotic cascade. On the contrary our anti-peptide antibodies are able to induce beta cell apoptosis upon engagment of a particular subtype of VGCC i.e CACNA1D. While dissecting the fine mechanisms by which our anti-peptide antibodies induce beta cell apoptosis is beyond the scope of the current study, we can hypothesyze two different scenarios.

First, our antibodies may be different from the ones described by Jackson et al. i.e. they react with CACNA1D, a particular subtype of VGCC, that we have identified; on the contrary the antibodies described by Jackson et al, react with a non better specified VGCC. It is possible that antibody activation of CACNA1D may by itself be sufficient to promote the entire apoptotic cascade. Second, the binding of our antibodies to the other identified beta cell antigens i.e. phogrin and PFKP, may provide the additional signals required to complete the entire apoptotic program. Binding of antibodies to phogrin might contribute to induce cellular stress by inducing a continuous insulin release even in absence of a natural stimulus such as glucose. Moreover the antibody binding to intracellular PKFP may lead to increased intracellular concentration of the activator enzyme F2-6BP and decresed production of ATP which may contribute to determine beta cell apoptosis. In this regard it is interesting to note that increased intracellular levels of F2-6BP have been found patients with diabetes [59].

### Conclusions

Type 1 diabetes is a chronic autoimmune disease characterized by progressive and selective destruction of pancreatic beta cells in genetically predisposed individuals. Beta cell apoptosis leading to progressive loss of pancreatic beta cells is a crucial step in the development of the disease without which clinical symptoms do not occurr. While the role of effector cells in beta-cell death is rather clearly defined, little is known on the role played by autoantibodies directed against beta-cell antigens. Among environmental factors, enteroviruses, in particular coxsackievirus B4, have been involved in the pathogenesis of type 1 diabetes. A direct link between autoantibodies, coxsackievirus B4 infection and beta-cell apoptosis in autoimmune diabetes has never been described. Our data indicate that in genetically predisposed individuals infection with coxsackievirus B4 is able to generate an anti-viral response able to be self-reactive towards beta-cell antigens through a molecular mimicry mechanism. Such (auto)antibodies induce beta-cell apoptosis through interaction with a particular type of voltage-gated calcium channel named CACNA1D. Our findings provide a previously unknown pathogenic mechanism by which coxsackievirus B4 infection is responsible for beta cells destruction in type 1 diabetes. To our knowledge our report represents the first description of a functional role of autoantibodies directed against beta cell antigens in type 1 diabetes. These findings beside providing important insights in disease pathogenesis may also represent important tools to generate alternative immuno-based therapheutical strategies for the treatment of early onset type 1 diabetes.

## Supporting Information

Figure S1 **Antibodies against T1DM, COXSA and CA peptides induce apoptosis in B-TC1 beta cell line.**
(TIF)

Table S1 **Clinical and laboratory features of the 58 patients enrolled for the screening of the peptide library.**
(DOC)

Table S2 **Homologies between human and murine beta-cell autoantigens.**
(PPT)

## References

[1] Eizirik DL, Colli ML, Ortis F (2009) The role of inflammation in insulitis andbeta cell loss in type 1 diabetes. Nat Rev Endocrinol 5: 219–226. 19352320 10.1038/nrendo.2009.21

[2] Richardson SJ, Willcox A, Bone AJ, Morgan NG, Foulis AK (2011) Immunopathology of the human pancreas in type-I diabetes. Semin Immunopathol 33: 9–21. 20424842 10.1007/s00281-010-0205-0

[3] Yeung WC, Rawlinson WD, Craig ME (2011) Enterovirus infection and type I diabetes mellitus: systematic review and meta-analysis of observational molecular studies. BMJ 342: d35 DOI10.1136/bmj.d35. 10.1136/bmj.d35 PMC3033438 21292721

[4] Stene LC, Rewers M (2012) Immunology in the clinic review series; focus on type 1 diabetes and viruses: the enterovirus link to type 1 diabetes: critical review of human studies. Clin Exp Immunol 168: 12–23. 22385232 10.1111/j.1365-2249.2011.04555.x PMC3390488

[5] Tauriainen S, Oikarinen S, Oikarinen M, Hyöty H (2011) Enteroviruses in the pathogenesis of type 1 diabetes. Semin Immunopathol 33: 45–55. 20424841 10.1007/s00281-010-0207-y

[6] Oikarinen S, Martiskainen M, Tauriainen S, Huhtala H, Ilonen J, et al (2011) Enterovirus RNA in blood is linked to the development of type 1 diabetes. Diabetes 60: 276–179. 20943747 10.2337/db10-0186 PMC3012181

[7] Schulte BM, Bakkers J, Lanke KH, Melchers WJ, Westerlaken C, et al (2010) Detection of enterovirus RNA in peripheral blood mononuclear cells of type 1 diabetic patients beyond the stage of acute infection.Viral Immunol. 23: 99–104. 10.1089/vim.2009.0072 20121407

[8] Hober D, Sauter P (2010) Pathogenesis of type I diabetes mellitus: interplay between enterovirus and host. Nat Rev Endocrinol 6: 279–289. 20351698 10.1038/nrendo.2010.27

[9] Oikarinen M, Tauriainen S, Oikarinen S, Honkanen T, Collin P, et al (2012) Type 1 diabetes is associated with enterovirus infection in gut mucosa. Diabetes 61: 687–691. 22315304 10.2337/db11-1157 PMC3282798

[10] Mercalli A, Lampasona V, Klingel K, Albarello L, Lombardoni C, et al (2012) No evidence of enteroviruses in the intestine of patients with type 1 diabetes. Diabetologia 55: 2479–2488. 22684312 10.1007/s00125-012-2591-4

[11] Dotta F, Censini S, van Halteren AG, Marselli L, Masini M, et al (2007) Coxsackie B4 virus infection of beta cells and natural killer cell insulitis in recent-onset type 1 diabetic patients. Proc Natl Acad Sci USA 104: 5115–5120. 17360338 10.1073/pnas.0700442104 PMC1829272

[12] Ylipaasto P, Klingel K, Lindberg AM, Otonkoski T, Kandolf R, et al (2004) Enterovirus infection in human pancreatic islet cells, islet tropism in vivo and receptor involvement in cultured islet beta cells. Diabetologia 47: 225–239. 14727023 10.1007/s00125-003-1297-z

[13] Grieco FA, Sebastiani G, Spagnuolo I, Patti A, Dotta F (2012) Immunology in the clinic review series; focus on type 1 diabetes and viruses: how viral infections modulate beta cell function. Clin Exp Immunol 168: 24–29. 22385233 10.1111/j.1365-2249.2011.04556.x PMC3390489

[14] Ghazarian L, Diana J, Simoni Y, Beaudoin L, Lehuen A (2012) Prevention or acceleration of type 1 diabetes by viruses. Cell Mol Life Sci DOI10.1007/s00018–012–1042–1. 10.1007/s00018-012-1042-1 PMC11113684 22766971

[15] Schulte BM, Lanke KH, Piganelli JD, Kers-Rebel ED, Bottino R, et al (2012) Cytokine and chemokine production by human pancreatic islets upon enterovirus infection. Diabetes 61: 2030–2036. 22596052 10.2337/db11-1547 PMC3402326

[16] Jaïdane H, Sané F, Hiar R, Goffard A, Gharbi J, et al (2012) Immunology in the clinic review series; focus on type 1 diabetes and viruses: enterovirus, thymus and type 1 diabetes pathogenesis. Clin Exp Immunol 168: 39–46. 22385235 10.1111/j.1365-2249.2011.04558.x PMC3390491

[17] Hober D, Sane F, Jaïdane H, Riedweg K, Goffard A, et al (2012) Immunology in the clinic review series; focus on type 1 diabetes and viruses: role of antibodies enhancing the infection with Coxsackievirus-B in the pathogenesis of type 1 diabetes. Clin Exp Immunol 168: 47–51. 22385236 10.1111/j.1365-2249.2011.04559.x PMC3390492

[18] In’t Veld P (2011) Insulitis in human type 1 diabetes: The quest for an elusive lesion. Islets 3: 131–138. 21606672 10.4161/isl.3.4.15728 PMC3154446

[19] Lunardi C, Bason C, Navone R, Millo E, Damonte G, et al (2000) Systemic sclerosis immunoglobulin G autoantibodies bind the human cytomegalovirus late protein UL94 and induce apoptosis in human endothelial cells. Nat Med 6: 1183–1186. 11017152 10.1038/80533

[20] Lunardi C, Bason C, Leandri M, Navone R, Lestani M, et al (2001) Autoantibodies to inner ear and endothelial antigens in Cogan syndrome. Lancet 360: 915–921. 10.1016/S0140-6736(02)11028-2 12354474

[21] Zanoni G, Navone R, Lunardi C, Tridente G, Bason C, et al (2006) In celiac disease, a subset of autoantibodies against transglutaminase binds toll-like receptor 4 and induces activation of monocytes. PLoS Med 3: e358. 16984219 10.1371/journal.pmed.0030358 PMC1569884

[22] Frulloni L, Lunardi C, Simone R, Dolcino M, Scattolini C, et al (2009) Identification of a novel antibody associated with autoimmune pancreatitis. N Engl J Med 361: 2135–2142. 19940298 10.1056/NEJMoa0903068

[23] Puccetti A, Lunardi C (2010) The role of peptide libraries in the identification of novel autoantigen targets in autoimmune diseases. Discov Med 9: 224–228. 20350489

[24] American Diabetes Association (2006) Diagnosis and classification of diabetes mellitus. Diabetes care S29: S43–S49.

[25] Wellings DA, Atherton E (1997) Standard FMOC protocols. Meth Enzymol 289: 44–67. 9353717 10.1016/s0076-6879(97)89043-x

[26] Troiano L, Ferraresi R, Lugli E, Nemes E, Roat E, et al (2007) Multiparametric analysis of cells with different mitochondrial membrane potential during apoptosis by polychromatic flow cytometry. Nat Protoc 2: 2719–2727. 18007607 10.1038/nprot.2007.405

[27] Cavalieri E, Rigo A, Bonifacio M, Carcereri de Prati A, Guardalben E, et al (2011) Pro-apoptotic activity of α-bisabolol in preclinical models of primary human acute leukemia cells. J Transl Med 21: 9–45. 10.1186/1479-5876-9-45 PMC3112094 21510902

[28] Pedemonte N, Boido D, Moran O, Giampieri M, Mazzei M, et al (2007) Structure-activity relationship of 1,4-dihydropyridines as potentiators of the cystic fibrosis transmembrane conductance regulator chloride channel. Mol Pharmacol 72: 197–207. 17452495 10.1124/mol.107.034702

[29] Richer MJ, Horwitz MS (2009) Coxsackievirus infection as an environmental factor in the etiology of type I diabetes. Autoimmun Rev 8: 601–615. 10.1016/j.autrev.2009.02.006 19393207

[30] Jaïdane H, Hober D (2008) Role of coxsackievirus B4 in the pathogenesis of type 1 diabetes. Diabetes Metabol 34: 537–548. 10.1016/j.diabet.2008.05.008 18951821

[31] Tuszkiewicz-Misztal E (1991) Epidemiologic factors and serum antibody titer to Coxsackie B4 virus in patients with type I diabetes. Kinderarztl Prax 59: 88–91. 1647469

[32] Jaïdane H, Sanè F, Gharbi J, Aouni M, Romond MB, et al (2009) Coxsackievirus B4 and type 1 diabetes pathogenesis: contribution of animal models. Diabetes Metab Res Rev 25: 591–603. 19621354 10.1002/dmrr.995

[33] Richarson SJ, Willicox A, Bone AJ, Foulis AK, Morgan NG (2009) The prevalence of enteroviral capsid protein VP1 immunostaining in pancreatic islets in human type 1 diabetes. Diabetologia 52: 1143–1151. 19266182 10.1007/s00125-009-1276-0

[34] Wasmeier C, Hutton JC (1996) Molecular cloning of phogrin, a protein tyrosine phosphatase homologue localized to insulin secretory granule membrane. J Biol Chem 271: 18161–18170. 8663434 10.1074/jbc.271.30.18161

[35] Kubosaki A, Gross S, Miura J, Saeki K, Zhu M, et al (2004) Targeted disruption of the IA-2beta gene causes glucose intolerance and impairs insulin secretion but does not prevent the development of diabetes in NOD mice. Diabetes 53: 1684–1691. 15220191 10.2337/diabetes.53.7.1684

[36] Torii S, Saito N, Kawano A (2009) Gene silencing of phogrin unveils its essential role in glucose-responsive pancreatic beta cell growth. Diabetes 58: 682–692. 19073770 10.2337/db08-0970 PMC2646067

[37] Torii S, Saito N, Kawano A, Zhao S, Izumi T, et al (2005) Cytoplasmic transport signal is involved in phogrin targeting and localization to secretory granules. Traffic 6: 1213–1224. 16262730 10.1111/j.1600-0854.2005.00353.x

[38] Kubosaki A, Nakamura S, Notkins AL (2006) Dense core vesicle proteins IA-2 and IA2beta metabolic alteration in double knockout mice. Diabetes 54: s46–s51. 10.2337/diabetes.54.suppl_2.s46 16306340

[39] Torii S (2009) Expression and function of IA-2 family proteins, unique neuroendocrine-specific protein-tyrosine phosphatases. Endocr J 56: 639–648. 19550073 10.1507/endocrj.k09e-157

[40] Sola-Penna M, Da Silva D, Coelho WS, Marinho-Carvalho MM, Zancan P (2010) Regulation of mammalian muscle type 6-phosphofructo-1-kinase and its implication for the control of the metabolism. IUBMB Life 62: 791–6. 21117169 10.1002/iub.393

[41] Coelho WS, Da Silva D, Marinho-Carvalho MM, Sola-Penna M (2012) Serotonin modulates hepatic 6-phosphofructo-1-kinase in an insulin synergistic manner. Int J Biochem Cell Biol 44: 150–157. 22037424 10.1016/j.biocel.2011.10.010

[42] Merrins MJ, Bertram R, Sherman A, Satin LS (2012) Phosphofructo-2-kinase/fructose-2,6-bisphosphatase modulates oscillations of pancreatic islet metabolism. PLoS One 7: e34. 10.1371/journal.pone.0034036 PMC3332096 22532827

[43] Ristow M, Vorgerd M, Möhlig M, Schatz H, Pfeiffer A (1999) Insulin resistance and impared insulin secretion due to phosphofructo-1-kinase-deficiency in humans. J Mol Med 77: 96–103. 9930938 10.1007/s001090050311

[44] Ristow M, Carlqvist H, Hebinck J (1999) Deficiency of phosphofructo-1-kinase/muscle subtype in humans is associated with impairment of insulin secretory oscillations. Diabetes 48: 1557–1561. 10426373 10.2337/diabetes.48.8.1557

[45] Yang SN, Berggren PO (2005) Beta Cell CaV channel regulation in physiology and pathophysiology. Am J Physiol Endocrinol Metab 288: E16–28. 15585596 10.1152/ajpendo.00042.2004

[46] Yang SN, Berggren PO (2006) The Role of Voltage-Gated Calcium Channels in Pancreatic Cell Physiology and Pathophysiology. Endocrine Rev 27: 621–676. 16868246 10.1210/er.2005-0888

[47] Juntti-Berggren L, Refai E, Appelskog I, Andersson M, Imreh G, et al (2004) Apolipoprotein CIII promotes Ca2+-dependent beta cell death in type 1 diabetes. Proc Natl Acad Sci U S A 101: 10090–10094. 15210953 10.1073/pnas.0403551101 PMC454169

[48] Wang L, Bhattacharjee A, Zuo Z, Hu F, Honkanen RE, et al (1999) A low voltage-activated Ca2+ current mediates cytokine-induced pancreatic beta-cell death. Endocrinology 140: 1200–1204. 10067844 10.1210/endo.140.3.6556

[49] Hell JW, Westenbroek RE, Warner C, Ahlijanian MK, Prystay W, et al (1993) Identification and differential subcellular localization of the neuronal class C and class D L-type calcium channel alpha 1 subunits. J Cell Biol 123: 949–962. 8227151 10.1083/jcb.123.4.949 PMC2200142

[50] Thomas HE, McKenzie MD, Angstetra E, Campbell PD, Kay TW (2009) Beta cell apoptosis in diabetes. Apoptosis 14: 1389–1404. 19322660 10.1007/s10495-009-0339-5

[51] McKenzie MD, Dudek NL, Mariana L, Chong MM, Trapani JA, et al (2006) Perforin and Fas induced by IFNgamma and TNFalpha mediate beta cell death by OT-I CTL. Int Immunol 18: 837–846. 16574667 10.1093/intimm/dxl020

[52] Thomas HE, Trapani JA, Kay TW (2010) The role of perforin and granzymes in diabetes. Cell Death Differ 17: 577–585. 19927156 10.1038/cdd.2009.165

[53] Chang I, Cho N, Kim S, Kim JY, Kim E, et al (2004) Role of calcium in pancreatic islet cell death by IFN-gamma/TNF-alfa. J Immunol 172: 7008–7014. 15153522 10.4049/jimmunol.172.11.7008

[54] Lee MS, Chang I, Kim S (2004) Death effectors of beta-cell apoptosis in type 1 diabetes. Mol Genet Metab 83: 82–92. 15464423 10.1016/j.ymgme.2004.08.002

[55] Lablanche S, Cottet-Rousselle C, Lamarche F, Benhamou PY, Halimi S, et al (2011) Protection of pancreatic INS-1 β-cells from glucose- and fructose-induced cell death by inhibiting mitochondrial permeability transition with cyclosporin A or metformin. Cell Death Dis 2: e134. 21430707 10.1038/cddis.2011.15 PMC3101812

[56] Galluzzi L, Zampami N, de La Motte Rouge T, Lemaire C, Brenner C, et al (2007) Methods for the assessment of mitochondrial membrane permeabilization in apoptosis. Apoptosis 12: 803–813. 17294081 10.1007/s10495-007-0720-1

[57] Colli ML, Nogueira TC, Allagnat F, Cunha DA, Gurzov EN, et al (2011) Exposure to the viral by-product dsRNA or Coxsackievirus B5 triggers pancreatic beta cell apoptosis via a Bim/Mcl-1 imbalance. PLoS Pathog 7: e1002267. 21977009 10.1371/journal.ppat.1002267 PMC3178579

[58] Jackson MW, Gordon TP (2010) A novel impedance-based cellular assay for the detection of anti-calcium channel autoantibodies in type 1 diabetes. J Immunol Methods 361: 31–36. 20655919 10.1016/j.jim.2010.07.005

[59] Atsumi T, Chiba H, Yoshioka N, Bucala R, Koike T (2007) Increased Fructose 2,6-bisphosphate in peripheral blood mononuclear cells of patients with diabetes. Endocr J 54: 517–520. 17510500 10.1507/endocrj.k06-205

