# Supplementary figures and images for: In Type 1 Diabetes a Subset of Anti-Coxsackievirus B4 Antibodies Recognize Autoantigens and Induce Apoptosis of Pancreatic Beta Cells
Source: PLoS One. 2013 Feb 28;8(2):e57729. doi: 10.1371/journal.pone.0057729 (PMC3585221; doi:10.1371/journal.pone.0057729)

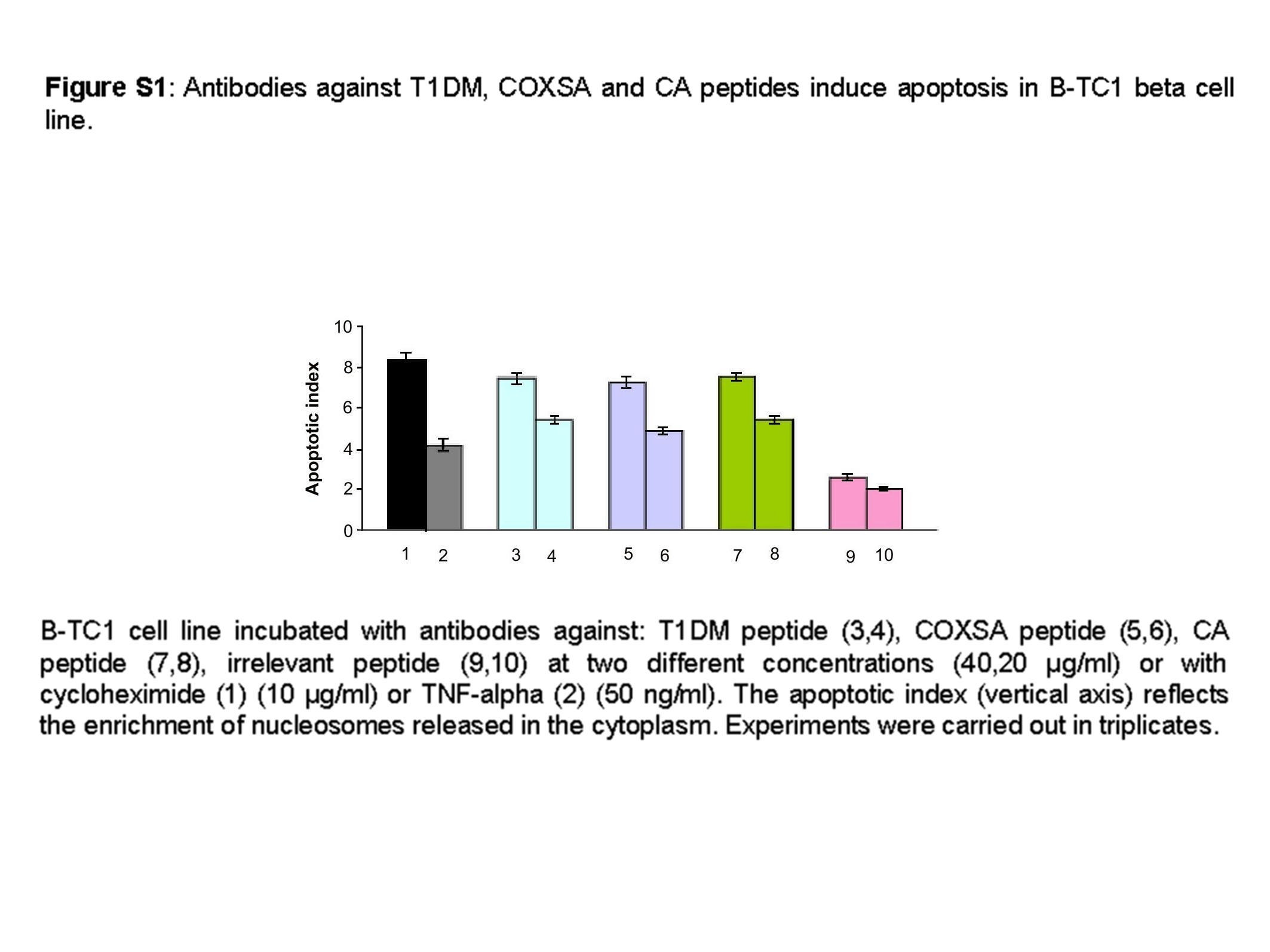

Supplement: Figure S1 — Antibodies against T1DM, COXSA and CA peptides induce apoptosis in B-TC1 beta cell line. (TIF) [file pone.0057729.s001.tif]
