# Supplementary material for: In Type 1 Diabetes a Subset of Anti-Coxsackievirus B4 Antibodies Recognize Autoantigens and Induce Apoptosis of Pancreatic Beta Cells
Source: PLoS One. 2013 Feb 28;8(2):e57729. doi: 10.1371/journal.pone.0057729 (PMC3585221; doi:10.1371/journal.pone.0057729)
Supplement: Table S1 — Clinical and laboratory features of the 58 patients enrolled for the screening of the peptide library. (DOC) [file pone.0057729.s002.doc]

**Table S1:** Clinical and laboratory features of the 58 patients enrolled for the screening of the peptide library

|  | **Patients** | **Gender (Males/ Females)** | **Age at T1DM diagnosis (Yrs)** | **Days after T1DM diagnosis** | **GADA**  **nv ≤ 1 AU** | **IA-2A**  **nv ≤ 1 AU** | **IAA**  **nv ≤ 10%** |
| --- | --- | --- | --- | --- | --- | --- | --- |
| 1 | B. F. | M | 12,2 | 1 | 6,0 | >100 | 5,5 |
| 2 | B. M. | M | 9,2 | 6 | 4,5 | <0,1 | 7,0 |
| 3 | B. F. | M | 0,8 | 1 | 29,0 | 0,7 | 16,9 |
| 4 | B. S. | F | 3,8 | 2 | 26,2 | 0,8 | 7,5 |
| 5 | B. F. | M | 11,6 | 22 | 1,4 | 8,3 | 12,7 |
| 6 | C. J. | F | 10,3 | 4 | 0,4 | 35,5 | 5,9 |
| 7 | C. A. | M | 13,7 | 3 | 119,3 | 10,2 | 1,9 |
| 8 | C. C. | F | 15,4 | 19 | 13,6 | 100,0 | 5,2 |
| 9 | C. M. | F | 16,8 | 2 | 5,2 | 8,6 | 7,7 |
| 10 | C. D. | M | 18,7 | 1 | 25,1 | 95,0 | 7,0 |
| 11 | C. C. | F | 9,8 | 1 | 3,5 | 1,0 | 4,9 |
| 12 | C. F. | F | 11,4 | 1 | 30,8 | 2,9 | 6,6 |
| 13 | D. R. | M | 10,4 | 4 | 5,4 | 0,0 | 10,1 |
| 14 | D. R. A. | M | 15,7 | 3 | 4,0 | 3,1 | 5,6 |
| 15 | D. P. A. | M | 5,4 | 2 | 0,0 | 2,6 | 6,7 |
| 16 | D. L. | M | 7,5 | 7 | 0,7 | 1,8 | 9,1 |
| 16 | F. C. | F | 15,3 | 2 | 14,0 | 52,5 | 4,6 |
| 18 | F. G. | F | 6,2 | 0 | 3,4 | 15,4 | 17,0 |
| 19 | G. F. | M | 8,4 | 1 | 0,1 | 15,6 | 5,5 |
| 20 | G. I. | F | 2,7 | 20 | 1,3 | 0,5 | 6,6 |
| 21 | G. M. | M | 9,3 | 0 | 10,2 | 10,6 | 5 |
| 22 | G. S. | F | 5,8 | 2 | 16,4 | 0,3 | 29,4 |
| 23 | I. M. | F | 8,0 | 1 | 0,0 | 2,6 | 1.5 |
| 24 | L. A. | M | 8,3 | 29 | 2,8 | >100 | 9,3 |
| 25 | L. F. | F | 3,3 | 6 | 0,0 | 2,1 | 6,6 |
| 26 | L. F. | M | 4,8 | 2 | 0,8 | >100 | 3 |
| 27 | L. C. | M | 10,1 | 4 | 37,2 | 0,3 | 8,2 |
| 28 | M. M. | F | 12,0 | 0 | >100 | >100 | 27 |
| 29 | M. I. | F | 8,9 | 1 | 1,7 | 5,6 | 6,8 |
| 30 | M. L. | M | 2,9 | 1 | 44,2 | 1,3 | 40,7 |
| 31 | M. S. | M | 14,1 | 4 | 28,3 | 12,3 | 5,6 |
| 32 | M. L. | M | 2,8 | 1 | 4,2 | 3,0 | 69,3 |
| 33 | M. S. | M | 13,6 | 7 | 1,5 | 24,1 | 1.5 |
| 34 | M. F. | M | 15,1 | 5 | 14,5 | 0,8 | 4,5 |
| 35 | N. F. | F | 9,6 | 0 | 0,7 | 58,4 | 6,8 |
| 36 | O. E. | F | 2,0 | 5 | 10,5 | >100 | 49,1 |
| 37 | P. L. | M | 12,3 | 2 | 1,6 | 32,0 | 5,8 |
| 38 | P. M. | M | 3,5 | 4 | 3,0 | <0,1 | 8,0 |
| 39 | P. S. | M | 10,2 | 2 | 1,1 | 27,0 | 8,2 |
| 40 | P. G. | F | 10,2 | 3 | 8,8 | <0.1 | 9,6 |
| 41 | P. A. | M | 4,4 | 6 | 1,1 | 2,7 | 4,7 |
| 42 | P. L. | M | 5,2 | 2 | 2,3 | 2,4 | 5,1 |
| 43 | P. I. | F | 3,7 | 1 | 59,4 | 0,6 | 3.2 |
| 44 | P. F. | M | 1,8 | 3 | 0,2 | 3,8 | 10,2 |
| 45 | R. M. | F | 12,5 | 23 | 4,3 | 47,0 | 4,9 |
| 46 | R. C. | M | 8,4 | 0 | 0,5 | 11,5 | 5,1 |
| 47 | R. A. | M | 14,2 | 1 | 4,8 | 19,8 | 3,2 |
| 48 | R. E. | M | 5,3 | 1 | 1,0 | 27,0 | 8,7 |
| 49 | R. R. | F | 5,3 | 0 | 0,0 | 2,8 | 8,6 |
| 50 | S. M. | M | 3,2 | 2 | 0,5 | 2,0 | 21,3 |
| 51 | S. A. | M | 3,0 | 1 | 56,9 | 1,0 | 50,5 |
| 52 | S. P. | M | 10,2 | 19 | 0,7 | 5,6 | 8,5 |
| 53 | T. S. | F | 7,1 | 4 | 49,6 | 0,1 | 3,5 |
| 54 | U. R. | M | 7,2 | 2 | 1,9 | <0,1 | 4,7 |
| 55 | V. C. | F | 4,5 | 0 | 36,7 | 21,2 | 7,7 |
| 56 | V. P. | M | 3,6 | 7 | 5,7 | 38,5 | 8,9 |
| 57 | V. M. | M | 2,9 | 2 | >100 | >100 | 7,6 |
| 58 | Z. J. | F | 8,5 | 5 | 21,0 | 43,5 | 6,0 |
|  | | | | | | | |

The presence of anti-glutamic acid decarboxylase (GADA), anti-tyrosine phosphatase-like protein (IA-2A) and anti-insulin (IAA) autoantibodies was evaluated with commercially available kits (Radioimmuno-assay CIS Bio International-Shering S.A.). Blood samples were obtained within 1-29 days after insulin therapy.

Positive samples (red values) were: 44/58 for GADA, 43/58 for IA-2A and 12/58 for IAA.
