# Supplementary material for: In Type 1 Diabetes a Subset of Anti-Coxsackievirus B4 Antibodies Recognize Autoantigens and Induce Apoptosis of Pancreatic Beta Cells
Source: PLoS One. 2013 Feb 28;8(2):e57729. doi: 10.1371/journal.pone.0057729 (PMC3585221; doi:10.1371/journal.pone.0057729)
Supplement: Table S2 — Homologies between human and murine beta-cell autoantigens. (PPT) [file pone.0057729.s003.ppt]

## Slide 1
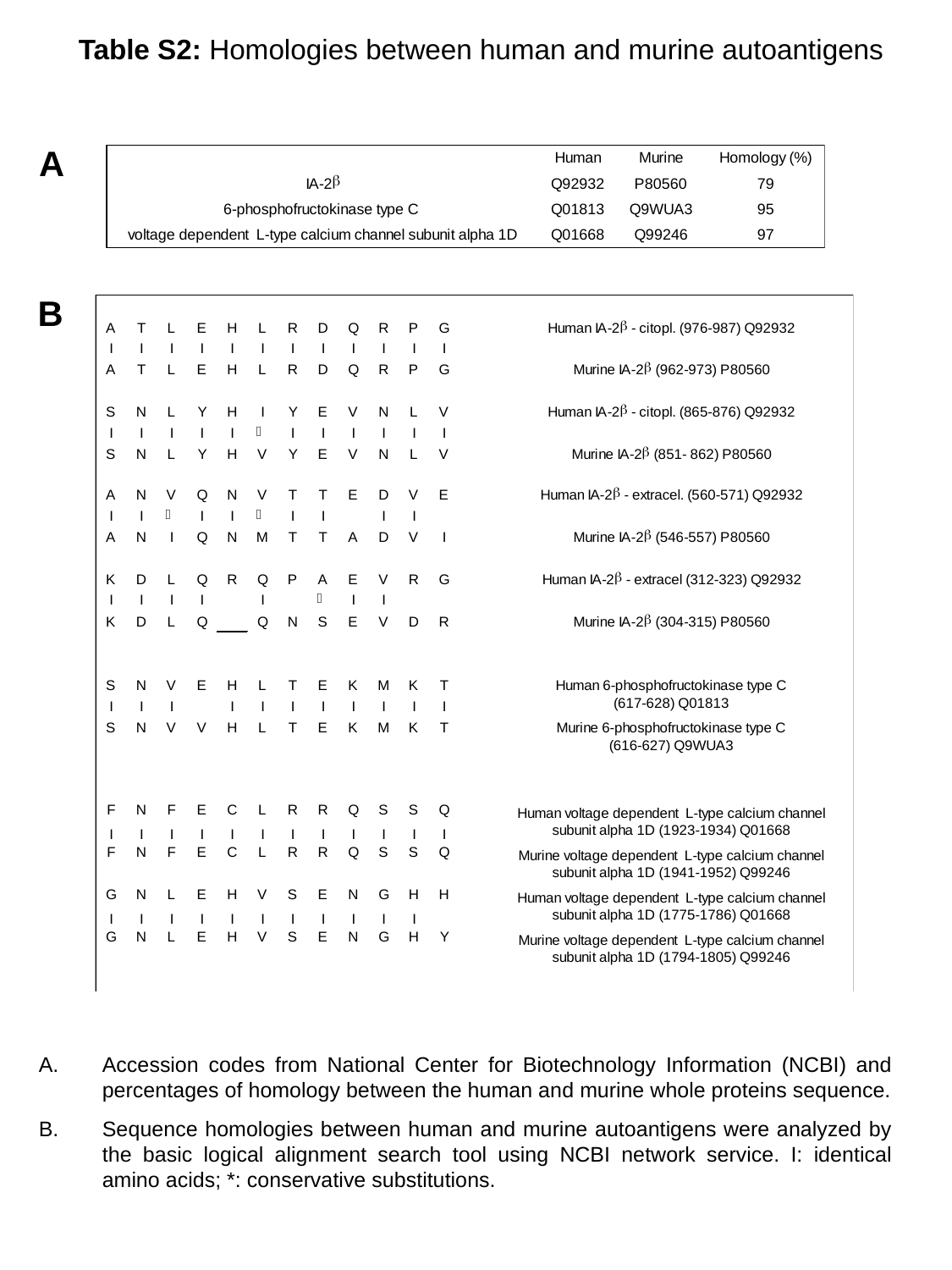

Table S2: Homologies between human and murine autoantigens
A
B
Accession codes from National Center for Biotechnology Information (NCBI) and percentages of homology between the human and murine whole proteins sequence.
Sequence homologies between human and murine autoantigens were analyzed by the basic logical alignment search tool using NCBI network service. I: identical amino acids; *: conservative substitutions.
